# Crystal structure of zwitterionic 4-(ammonio­methyl)­benzoate: a simple mol­ecule giving rise to a complex supra­molecular structure

**DOI:** 10.1107/S1600536814022831

**Published:** 2014-10-24

**Authors:** Ana María Atria, Maria Teresa Garland, Ricardo Baggio

**Affiliations:** a Facultad de Ciencias Químicas y Farmacéuticas, Universidad de Chile, Casilla 233, Santiago, Chile; b Departamento de Física, Facultad de Ciencias Físicas y Matemáticas, Universidad de Chile, Santiago de Chile, Chile; c Departamento de Física, Centro Atómico Constituyentes, Comisión Nacional de Energía Atómica, Buenos Aires, Argentina

**Keywords:** crystal structure, zwitterion, crystal packing, 4-(ammonio­meth­yl)benzoate, N—H⋯O hydrogen bonds, π–π stacking

## Abstract

The asymmetric unit of the title compound, C_8_H_9_NO_2_·H_2_O consists of an isolated 4-(ammonio­meth­yl)benzoate zwitterion derived from 4-amino­methyl­benzoic acid through the migration of the acidic proton, together with a water molecule of crystallization that is disordered over three sites with occupancy ratios (0.50:0.35:0.15). In the crystal structure, N—H⋯O hydrogen bonds together with π–π stacking of the benzene rings [centroid–centroid distance = 3.8602 (18) Å] result in a strongly linked, compact three-dimensional structure.

## Abstract 2

The asymmetric unit consists of an isolated 4-(ammonio­meth­yl)benzoate zwitterion derived from 4-amino­methyl­benzoic acid through the migration of the acidic proton, together with a solvate water that is disordered over three sites. In the crystal structure, N—H⋯O hydrogen bonds together with π–π stacking of the benzene rings result in a strongly linked, compact three-dimensional structure.

## Chemical context   

As part of a long-range project to find new transition-metal complexes of simple ligands such as carboxyl­ates and amines, we have screened a number of derivatives of benzoic acid, in particular those that a search of the Cambridge Structural Database (CSD, Version 5.35, updated to May 2014; Groom & Allen, 2014 ▶) reveals to have formed few coordination complexes whose structures have been reported. The title compound was the unexpected product of an attempt to form a Co^II^ complex with 4-amino­methyl­benzoic acid [HAMBA, (*a*) in scheme below], which has no entries in the CSD, and di­amino­purine (DAP).
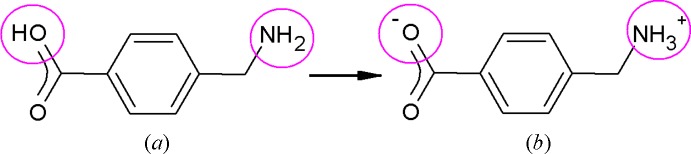


No coordination complex resulted, but the reaction provided, as an unexpected bonus, a crystalline phase of the monohydrate of the zwitterion of HAMBA (see scheme below), in which the acidic proton has migrated to the amino group resulting in COO^−^ and CH_2_NH_3_^+^ substituents on the aromatic ring and forming 4-(ammonio­meth­yl)benzoate [(*b*) in scheme above]. In contrast to the utmost simplicity of its mol­ecular structure, the zwitterion displays an extremely complex hydrogen-bonding scheme and concomitant supra­molecular structure as reported herein.
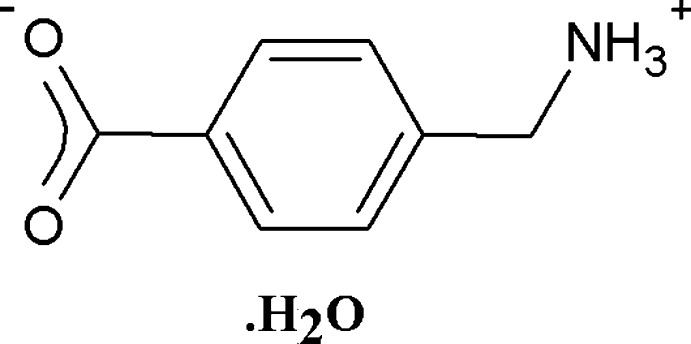


## Structural commentary   

**Figure 1:**
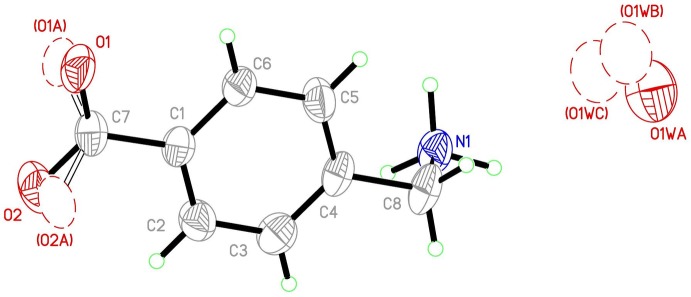
The asymmetric unit of (I). The minor disorder component of the carboxyl­ate group and those of the solvate water mol­ecule are drawn with broken lines.

Fig. 1 ▶ shows the asymmetric unit of the title compound, (I). The C—C_6_—C backbone is essentially planar [maximum deviation of 0.005 (3) Å for C8], and subtends dihedral angles of 6.8 (2) and 83.9 (2)° with the O_2_C–C (major disorder component) and C–CN planes, respectively. Bond lengths and angles are normal, with the C—O bond lengths of the carboxyl­ate group close to equal, indicating extensive electron delocalization over the unit [C7—O1: 1.266 (4), C7—O2: 1.262 (4) Å].

## Supra­molecular features   

**Figure 2:**
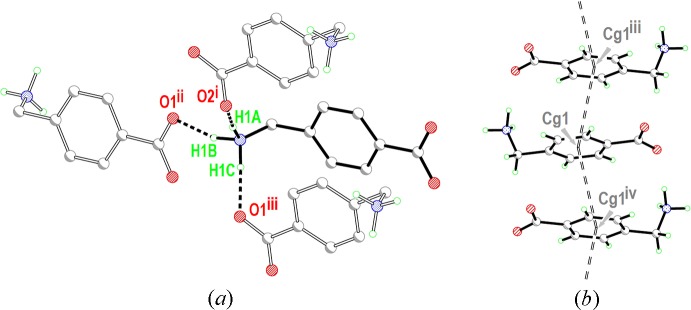
(*a*) Hydrogen-bonding and (*b*) π–π inter­actions in (I). Symmetry codes: (i) *x* − 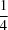, −*y* + 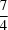, *z* − 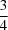; (ii) −*x* + 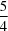, *y* − 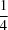, *z* − 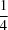; (iii) *x* − 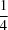, −*y* + 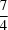, *z* + 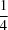; (iv) *x* + 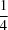, −*y* + 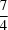, *z* − 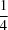.

**Figure 3:**
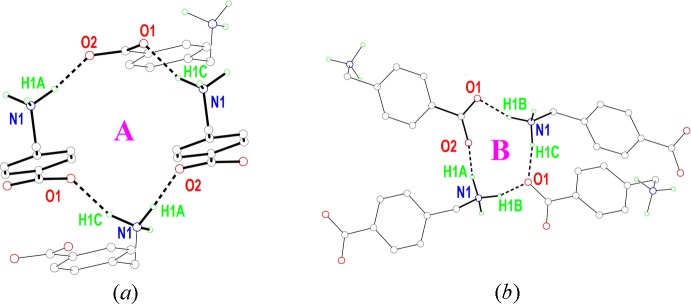
(*a*) 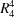
(24) loops, A, formed by mol­ecules of (I) through N1—H1*A*⋯O2 and N1—H1*C*⋯O1 hydrogen bonds. (*b*) 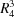
(10) loops, B, formed by mol­ecules of (I) through N—H⋯O contacts involving all three H atoms of the NH_3_^+^ substituent.

As indicated previously, the most inter­esting features in the structure are those derived from the inter­molecular inter­actions, presented in Table 1 ▶ (hydrogen bonds) and Table 2 ▶ (π–π contacts). Each ammonium group is bound through N—H⋯O hydrogen bonds to three different mol­ecules of (I), with the carboxyl­ato oxygen atoms as acceptors (Fig. 2 ▶ *a*). In addition, the benzene rings stack almost parallel to each other in slanted columns (Fig. 2 ▶ *b*). N1—H1*A*⋯O2 and N1—H1*C*⋯O1 hydrogen bonds link four mol­ecules together, generating 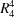
(24) ring motifs, Fig. 3 ▶ *a*, while a second synthon with an 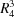
(10) graph set motif is generated through contacts involving all three hydrogens of the ammonium cation, Fig. 3 ▶ *b* (for graph-set notation see, for example, Bernstein *et al.*, 1995 ▶).

**Figure 4:**
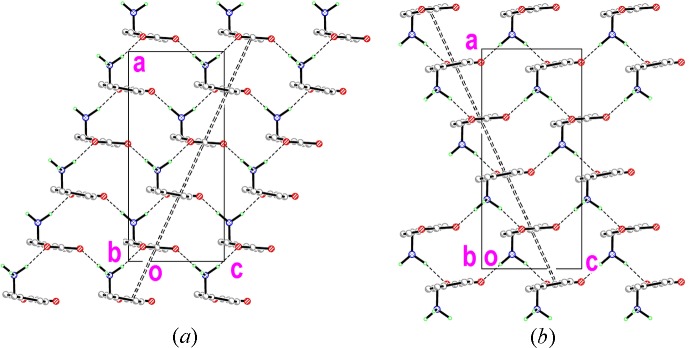
Sheets of mol­ecules of (I) in the *ac* plane linked by N—H⋯O hydrogen bonds (single dashed lines) and π–π inter­actions (double dashed lines).

The 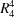
(24) synthons combine with the π–π stacking inter­actions to generate layers of mol­ecules in the *ac* plane. The π–π contacts are inclined parallel to either the (101) plane for one set of contacts (Fig. 4 ▶ *a*) or the (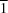
01) plane for the other (Fig. 4 ▶ *b*).

**Figure 5:**
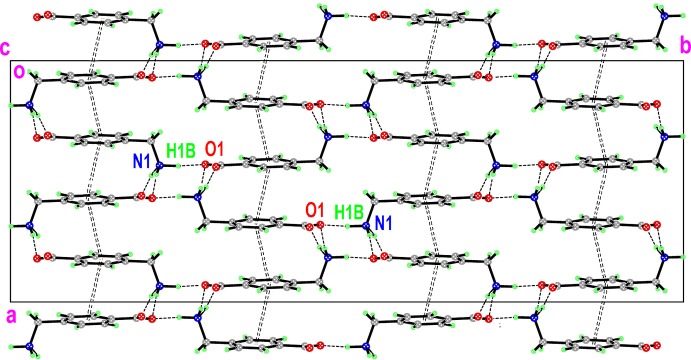
Chains of mol­ecules of (I) linked by N—H⋯O hydrogen bonds to form a three-dimensional network.

Fig. 5 ▶ shows a view along the *c* axis, and reveals the ‘corrugated’ shape of these sheets, consisting of zigzag chains of mol­ecules linked in a head-to-tail fashion and stacked roughly along the *a-*axis direction. Adjacent sheets are inter­connected along *b* in an obverse fashion by N1—H1*B*⋯O1 hydrogen bonds.

**Figure 6:**
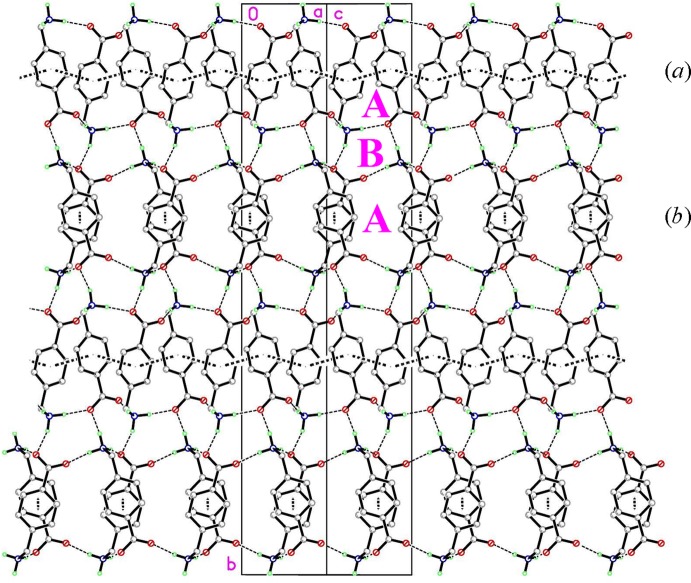
Overall packing for (I) showing how the A and B ring motifs combine with π–π stacking inter­actions to generate a three-dimensional network.

Finally, Fig. 6 ▶ presents a view approximately along the *ac* diagonal displaying the two hydrogen-bonding synthons, A and B, together with the π–π inter­actions and demonstrates how they combine to generate the three-dimensional network.

## Database survey   

Neither 4-(ammonio­meth­yl)benzoate nor its zwitterionic form described here appear in the CSD (Version 5.35, updated to May 2014). The most closely related structures are those of a zwitterionic form of 4-ammonio­methyl­cyclo­hexane-1-carb­oxy­lic acid (II*a*) (Shahzadi *et al.*, 2007 ▶; CSD refcode AMMCHC11) and its hemihydrated analogue (II*b*) (Yamazaki *et al.*, 1981 ▶; CSD refcode AMCHCA), in which the phenyl ring is replaced by cyclo­hexane. This introduces some obvious differences with (I), for π–π contacts are clearly precluded and there are different relative orientations of the hydrogen-bonding donors and acceptors. In spite of this, the hydrogen-bonding schemes do show some striking similarities, leading to similar (though differently connected) two-dimensional sub-structures. In particular, the same 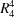
(24) and 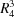
(10) synthons are present in both cases as in (I), and play predominant roles in the crystal packing. This is despite the presence of the water solvate in (II*b*), which is not involved in classical hydrogen bonding to the zwitterion.

## Synthesis and crystallization   

To an aqueous solution of HAMBA (1 mmol, 0.15116g) were added an aqueous solution of Co(Ac)_2_·4H_2_O (2 mmol, 0.49816g) and an ethano­lic solution of DAP (1 mmol, 0.15009 g). The resulting mixture was heated at reflux for 4 h and left to cool down and evaporate at room temperature. After a few days, crystals suitable for X-ray diffraction of the (uncomplexed) zwitterion (I) appeared. These were used as grown.

## Refinement   

Crystal data, data collection and structure refinement details are summarized in Table 3 ▶.

There are two disorder features in this structure. The oxygen atoms of the carboxyl­ate group were disordered over two positions that were refined with similarity restraints with occupancy factors 0.912 (13)/0.088 (13). Disorder involving the water molecule was more pronounced, with the oxygen atoms disordered over three distinct sites. When refined, the occupancies appeared to be strongly correlated with their displacement factors, showing an oscillating behaviour. In the final refinement cycles, occupancies were fixed to the mean values of these oscillation ranges with occupancy ratios 0.50:0.35:0.15.

All the H atoms (except for those of the disordered water mol­ecules) were recognizable in an early difference Fourier map. Hydrogen atoms of the NH_3_ group were refined with N—H distances restrained to be equal to within 0.01Å [final d(N—H) = 1.07 (3) Å]. All H atoms bound to carbon were refined using a riding model with *d*(C—H) = 0.93 Å and *U*_iso_ = 1.2*U*_eq_(C) for aromatic and 0.98 Å, *U*_iso_ = 1.2*U*_eq_(C) for methyl­ene H atoms. The hydrogen atoms on the disordered water solvate were not identified.

When trying to calculate the Flack parameter of the inverted structure, it was recognised that the space group was one of the few (seven, in fact) where the structure cannot be inverted by simple inversion of the atomic coordinates. In the case of *Fdd*2, the ‘inversion rule’ to be applied is Inv(*x*, *y*, *z*) = 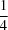 − *x*, 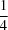 − *y*, −*z*, After this, the refinement proceeded smoothly without any change in the symmetry operators. Even so, the resulting Flack Parameters were both disparate and high [−1.2 (4) *vs* 2.2 (4) for the reported/inverted structures, respectively]. Hence, the absolute configuration could not be determined reliably.

## Supplementary Material

Crystal structure: contains datablock(s) I, global. DOI: 10.1107/S1600536814022831/sj5430sup1.cif

Structure factors: contains datablock(s) I. DOI: 10.1107/S1600536814022831/sj5430Isup2.hkl

Supporting information file. DOI: 10.1107/S1600536814022831/sj5430Isup3.cml

CCDC reference: 1029721

Additional supporting information:  crystallographic information; 3D view; checkCIF report

## Figures and Tables

**Table 1:** Hydrogen-bond geometry (, )

*D*H*A*	*D*H	H*A*	*D* *A*	*D*H*A*
N1H1*A*O2^i^	1.07(3)	1.75(3)	2.804(4)	170(3)
N1H1*B*O1^ii^	1.07(3)	1.73(4)	2.768(3)	162(4)
N1H1*C*O1^iii^	1.07(3)	1.87(3)	2.901(6)	161(3)

Symmetry codes: (i) 
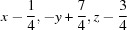
; (ii) 
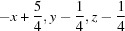
; (iii) 
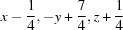
.

**Table 2:** contacts (, ) *Cg*1 is the centroid of atoms C1C6. ccd is the centroidcentroid distance, da is the dihedral angle between rings and ipd is the interplanar distance, or (mean) distance from one plane to the neighbouring centroid. For details, see Janiak (2000 ▶).

Group 1Group 2	ccd	da	ipd
*Cg*1*Cg*1^iii^	3.8602(18)	0.7 (2)	3.665(5)

Symmetry code: (iii) *x* 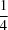, *y*+7/4, *z*+1/4.

**Table 3:** Experimental details

Crystal data	
Chemical formula	C_8_H_9_NO_2_H_2_O
*M*_r_	169.18
Crystal system, space group	Orthorhombic, *F* *d* *d*2
Temperature (K)	297
*a*, *b*, *c* ()	13.743(3), 38.302(7), 6.2686(11)
*V* (^3^)	3299.7(11)
*Z*	16
Radiation type	Mo *K*
(mm^1^)	0.11
Crystal size (mm)	0.48 0.30 0.22

Data collection	
Diffractometer	Bruker *SMART* CCD area detector
Absorption correction	Multi-scan (*SADABS*; Bruker, 2002 ▶)
*T*_min_, *T*_max_	0.94, 0.98
No. of measured, independent and observed [*I* > 2(*I*)] reflections	6720, 1827, 1555
*R*_int_	0.021
(sin /)_max_ (^1^)	0.659

Refinement	
*R*[*F* ^2^ > 2(*F* ^2^)], *wR*(*F* ^2^), *S*	0.046, 0.137, 1.04
No. of reflections	1827
No. of parameters	134
No. of restraints	13
H-atom treatment	H atoms treated by a mixture of independent and constrained refinement
_max_, _min_ (e ^3^)	0.21, 0.18
Absolute structure	Flack *x* determined using 616 quotients [(*I* ^+^)(*I* )]/[(*I* ^+^)+(*I* )] (Parsons Flack, 2004 ▶)
Absolute structure parameter	1.2(4)

Computer programs: *SMART* (Bruker, 2001 ▶) and *SAINT* (Bruker, 2002 ▶), *SHELXS97*, *SHELXL2014* and *SHELXTL* (Sheldrick, 2008 ▶) and *PLATON* (Spek, 2009 ▶).

## supplementary crystallographic information

### Crystal data

**Table:**

C_8_H_9_NO_2_·H_2_O	*D*_x_ = 1.362 Mg m^−^^3^
*M**_r_* = 169.18	Mo *K*α radiation, λ = 0.71073 Å
Orthorhombic, *F**d**d*2	Cell parameters from 1680 reflections
*a* = 13.743 (3) Å	θ = 3.1–26.1°
*b* = 38.302 (7) Å	µ = 0.11 mm^−^^1^
*c* = 6.2686 (11) Å	*T* = 297 K
*V* = 3299.7 (11) Å^3^	Block, pale pink
*Z* = 16	0.48 × 0.30 × 0.22 mm
*F*(000) = 1440	

### Data collection

**Table:**

Bruker SMART CCD area detector diffractometer	1555 reflections with *I* > 2σ(*I*)
CCD rotation images, thin slices scans	*R*_int_ = 0.021
Absorption correction: multi-scan (*SADABS*; Bruker, 2002)	θ_max_ = 27.9°, θ_min_ = 2.1°
*T*_min_ = 0.94, *T*_max_ = 0.98	*h* = −16→18
6720 measured reflections	*k* = −48→47
1827 independent reflections	*l* = −8→8

### Refinement

**Table:**

Refinement on *F*^2^	Hydrogen site location: mixed
Least-squares matrix: full	H atoms treated by a mixture of independent and constrained refinement
*R*[*F*^2^ > 2σ(*F*^2^)] = 0.046	*w* = 1/[σ^2^(*F*_o_^2^) + (0.0839*P*)^2^ + 1.177*P*] where *P* = (*F*_o_^2^ + 2*F*_c_^2^)/3
*wR*(*F*^2^) = 0.137	(Δ/σ)_max_ < 0.001
*S* = 1.04	Δρ_max_ = 0.21 e Å^−^^3^
1827 reflections	Δρ_min_ = −0.18 e Å^−^^3^
134 parameters	Absolute structure: Flack x determined using 616 quotients [(*I*^+^)-(*I*^-^)]/[(*I*^+^)+(*I*^-^)] (Parsons & Flack, 2004)
13 restraints	Absolute structure parameter: −1.2 (4)

### Special details

**Table:**

Geometry. All e.s.d.'s (except the e.s.d. in the dihedral angle between two l.s. planes) are estimated using the full covariance matrix. The cell e.s.d.'s are taken into account individually in the estimation of e.s.d.'s in distances, angles and torsion angles; correlations between e.s.d.'s in cell parameters are only used when they are defined by crystal symmetry. An approximate (isotropic) treatment of cell e.s.d.'s is used for estimating e.s.d.'s involving l.s. planes.

### Fractional atomic coordinates and isotropic or equivalent isotropic displacement parameters (Å^2^)

**Table:**

	*x*	*y*	*z*	*U*_iso_*/*U*_eq_	Occ. (<1)
O1	0.6801 (4)	0.96039 (6)	0.9009 (5)	0.0628 (10)	0.912 (13)
O2	0.6899 (4)	0.94439 (9)	1.2429 (5)	0.0759 (12)	0.912 (13)
O1A	0.641 (3)	0.9615 (6)	0.946 (6)	0.0628 (10)	0.088 (13)
O2A	0.736 (3)	0.9413 (8)	1.210 (5)	0.0759 (12)	0.088 (13)
C1	0.67898 (18)	0.89967 (7)	0.9810 (4)	0.0461 (6)	
C2	0.6919 (2)	0.87399 (7)	1.1326 (5)	0.0579 (7)	
H2	0.7033	0.8802	1.2738	0.069*	
C3	0.6881 (2)	0.83912 (7)	1.0764 (6)	0.0620 (8)	
H3	0.6974	0.8221	1.1800	0.074*	
C4	0.6705 (2)	0.82944 (6)	0.8686 (5)	0.0507 (7)	
C5	0.6570 (2)	0.85503 (7)	0.7172 (5)	0.0584 (7)	
H5	0.6449	0.8488	0.5764	0.070*	
C6	0.6612 (2)	0.89013 (7)	0.7727 (5)	0.0538 (7)	
H6	0.6521	0.9072	0.6691	0.065*	
C7	0.68372 (16)	0.93753 (7)	1.0465 (5)	0.0546 (7)	
C8	0.6665 (2)	0.79104 (7)	0.8069 (7)	0.0653 (9)	
H8A	0.6956	0.7880	0.6672	0.078*	
H8B	0.7040	0.7775	0.9085	0.078*	
N1	0.5655 (2)	0.77823 (6)	0.8030 (5)	0.0576 (6)	
H1A	0.524 (2)	0.7907 (12)	0.682 (7)	0.131 (18)*	
H1B	0.5636 (19)	0.7506 (9)	0.776 (8)	0.119 (16)*	
H1C	0.529 (2)	0.7830 (10)	0.951 (6)	0.16 (2)*	
O1WA	0.6665 (7)	0.73276 (19)	0.2872 (17)	0.100 (2)	0.5
O1WB	0.6438 (12)	0.7488 (3)	0.244 (3)	0.100 (2)	0.35
O1WC	0.647 (2)	0.7552 (7)	0.323 (5)	0.100 (2)	0.15

### Atomic displacement parameters (Å^2^)

**Table:**

	*U*^11^	*U*^22^	*U*^33^	*U*^12^	*U*^13^	*U*^23^
O1	0.088 (3)	0.0314 (9)	0.0690 (15)	−0.0019 (11)	0.0092 (15)	−0.0041 (10)
O2	0.107 (3)	0.0553 (14)	0.0655 (15)	−0.0144 (17)	−0.0026 (16)	−0.0195 (12)
O1A	0.088 (3)	0.0314 (9)	0.0690 (15)	−0.0019 (11)	0.0092 (15)	−0.0041 (10)
O2A	0.107 (3)	0.0553 (14)	0.0655 (15)	−0.0144 (17)	−0.0026 (16)	−0.0195 (12)
C1	0.0512 (14)	0.0333 (12)	0.0537 (15)	−0.0039 (9)	0.0023 (11)	−0.0037 (10)
C2	0.0784 (19)	0.0426 (14)	0.0526 (16)	−0.0071 (13)	−0.0054 (16)	−0.0023 (12)
C3	0.0843 (19)	0.0379 (13)	0.0638 (19)	−0.0031 (14)	−0.0036 (14)	0.0076 (13)
C4	0.0527 (13)	0.0296 (11)	0.0697 (17)	−0.0005 (10)	0.0082 (12)	−0.0056 (12)
C5	0.083 (2)	0.0411 (14)	0.0511 (15)	−0.0050 (13)	0.0033 (14)	−0.0103 (12)
C6	0.0757 (18)	0.0318 (12)	0.0538 (16)	−0.0001 (11)	−0.0040 (13)	−0.0002 (11)
C7	0.0639 (16)	0.0372 (13)	0.0626 (18)	−0.0062 (11)	0.0025 (13)	−0.0113 (13)
C8	0.0710 (18)	0.0320 (12)	0.093 (2)	0.0035 (12)	0.0091 (17)	−0.0108 (14)
N1	0.0777 (15)	0.0303 (10)	0.0648 (15)	−0.0039 (10)	−0.0015 (12)	−0.0033 (11)
O1WA	0.102 (4)	0.086 (5)	0.110 (6)	−0.028 (5)	0.013 (4)	−0.009 (5)
O1WB	0.102 (4)	0.086 (5)	0.110 (6)	−0.028 (5)	0.013 (4)	−0.009 (5)
O1WC	0.102 (4)	0.086 (5)	0.110 (6)	−0.028 (5)	0.013 (4)	−0.009 (5)

### Geometric parameters (Å, º)

**Table:**

O1—C7	1.266 (4)	C4—C5	1.377 (4)
O2—C7	1.262 (4)	C4—C8	1.521 (4)
O1A—C7	1.259 (13)	C5—C6	1.390 (4)
O2A—C7	1.256 (13)	C5—H5	0.9300
C1—C6	1.378 (4)	C6—H6	0.9300
C1—C2	1.379 (4)	C8—N1	1.472 (4)
C1—C7	1.509 (4)	C8—H8A	0.9700
C2—C3	1.382 (4)	C8—H8B	0.9700
C2—H2	0.9300	N1—H1A	1.07 (3)
C3—C4	1.376 (5)	N1—H1B	1.07 (3)
C3—H3	0.9300	N1—H1C	1.07 (3)
			
C6—C1—C2	119.1 (2)	O2A—C7—O1A	126.0 (16)
C6—C1—C7	121.4 (2)	O2—C7—O1	124.2 (3)
C2—C1—C7	119.5 (3)	O2A—C7—C1	111.0 (15)
C1—C2—C3	120.6 (3)	O1A—C7—C1	123.0 (14)
C1—C2—H2	119.7	O2—C7—C1	118.0 (3)
C3—C2—H2	119.7	O1—C7—C1	117.8 (3)
C4—C3—C2	120.6 (3)	N1—C8—C4	111.1 (2)
C4—C3—H3	119.7	N1—C8—H8A	109.4
C2—C3—H3	119.7	C4—C8—H8A	109.4
C3—C4—C5	119.0 (2)	N1—C8—H8B	109.4
C3—C4—C8	120.5 (3)	C4—C8—H8B	109.4
C5—C4—C8	120.5 (3)	H8A—C8—H8B	108.0
C4—C5—C6	120.7 (3)	C8—N1—H1A	111.9 (15)
C4—C5—H5	119.6	C8—N1—H1B	110.8 (15)
C6—C5—H5	119.6	H1A—N1—H1B	109 (4)
C1—C6—C5	120.1 (3)	C8—N1—H1C	111.6 (16)
C1—C6—H6	120.0	H1A—N1—H1C	107 (2)
C5—C6—H6	120.0	H1B—N1—H1C	107 (2)

### Hydrogen-bond geometry (Å, º)

**Table:**

*D*—H···*A*	*D*—H	H···*A*	*D*···*A*	*D*—H···*A*
N1—H1*A*···O2^i^	1.07 (3)	1.75 (3)	2.804 (4)	170 (3)
N1—H1*B*···O1^ii^	1.07 (3)	1.73 (4)	2.768 (3)	162 (4)
N1—H1*C*···O1^iii^	1.07 (3)	1.87 (3)	2.901 (6)	161 (3)

Symmetry codes: (i) *x*−1/4, −*y*+7/4, *z*−3/4; (ii) −*x*+5/4, *y*−1/4, *z*−1/4; (iii) *x*−1/4, −*y*+7/4, *z*+1/4.
